# Quinacrine directly dissociates amyloid plaques in the brain of 5XFAD transgenic mouse model of Alzheimer’s disease

**DOI:** 10.1038/s41598-021-91563-y

**Published:** 2021-06-08

**Authors:** Sohui Park, Hye Yun Kim, Hyun-A Oh, Jisu Shin, In Wook Park, Soljee Yoon, Dong Ho Woo, YoungSoo Kim

**Affiliations:** 1 Department of Pharmacy, Department of Integrative Biotechnology and Translational Medicine, and Yonsei Institute of Pharmaceutical Sciences, Yonsei University, Incheon, 21983 Republic of Korea; 2 Research Center for Convergence Toxicology, Korea Institute of Toxicology, Daejeon, 34114 Republic of Korea

**Keywords:** Small molecules, Alzheimer's disease

## Abstract

Alzheimer’s disease (AD) is the most common type of dementia characterized by the abnormal accumulation of amyloid-β (Aβ) in the brain. Aβ misfolding is associated with neuroinflammation and synaptic dysfunction, leading to learning and memory deficits. Therefore, Aβ production and aggregation have been one of the most popular drug targets for AD. Failures of drug candidates regulating the aforementioned Aβ cascade stimulated development of immunotherapy agents for clearance of accumulated Aβ in the brain. Here, we report that quinacrine, a blood–brain barrier penetrating antimalarial chemical drug, dissociates Aβ plaques in the brain of AD transgenic mice. When co-incubated with pre-formed Aβ fibrils, quinacrine decreased thioflavin T-positive β-sheets in vitro, on top of its inhibitory function on the fibril formation. We confirmed that quinacrine induced dissociation of high-molecular-weight Aβ aggregates into low-molecular-weight species by dot blots in association with size cut-off filtrations. Quinacrine was then administered to adult 5XFAD transgenic mice via weekly intravenous injections for 6 weeks, and we found a significant reduction of Aβ plaques and astrocytosis in their cortex and hippocampus. In western blots of quinacrine-administered mouse brains, amelioration of AD-related biomarkers, glial fibrillary acidic protein, postsynaptic protein 95, phosphorylated cAMP response element-binding protein, phosphorylated c-Jun N-terminal kinase were observed. Lastly, quinacrine-stimulated dissociation of misfolded aggregates induced recovery of synaptic function associated with Aβ in excitatory post-synaptic current recordings of primary rat cortical neurons treated with Aβ aggregates and quinacrine. Collectively, quinacrine can directly dissociate Aβ fibrils and alleviate decreased synaptic functions.

## Introduction

Alzheimer’s disease (AD) is a progressive neurodegenerative disorder characterized by misfolded amyloid-β (Aβ) in the brain. Aggregated Aβ induces astrocytosis and synaptic impairments in brain regions responsible for learning and memory^1,2^. As Aβ deposition is strongly correlated with the onset of AD, there has been an active search for chemical and biological agents that regulate Aβ pathology for the treatment of AD^3,4^. Considering the continuous failure of drug candidates inhibiting Aβ production and aggregation in clinical approval, the latest drug discovery has shifted its target to clearance of accumulated Aβ in the brain, mainly by immunotherapy^5,6^. Although the correlation of Aβ clearance and cognitive recovery is not clearly understood yet, lowering the Aβ aggregates associated with AD pathogenesis is considered a prominent therapeutic approach^4,7–9^.

In this study, we tested the possible drug repositioning of quinacrine, an FDA-approved antimalarial drug, for AD^10^. Previously, quinacrine was found to prevent the conversion of the normal host prion (PrP^C^) to disease-causing prion isoform (PrP^Sc^) by binding and stabilizing the PrP^C^^11,12^. We hypothesized that quinacrine may bind to misfolding proteins and tested its possible anti-aggregation function in Aβ-induced AD models. Utilizing the fluorescence thioflavin T (ThT) dye and molecular weight cut-off (MWCO) followed by anti-Aβ dot blots, we monitored the change of Aβ fibril formation and found that quinacrine inhibited and reversed the protein misfolding process. To study how quinacrine affects Aβ deposition and AD-associated protein levels in vivo, we intravenously injected quinacrine (2 mg/kg) to 7-month-old male 5XFAD mice, and the alterations in the levels of the protein biomarkers in the brain were analyzed through immunohistochemistry and western blot methods. To test if quinacrine-induced dissociation of Aβ aggregates ameliorates impaired synaptic functions, we cultured rat primary cortical neurons, exposed them to Aβ aggregates, and measured excitatory pre-synaptic current (EPSC) depending on the quinacrine treatment.

## Methods

### Reagents

Aβ(1–42) peptides were synthesized using solid-phase peptide synthesis as previously reported^13^. Thioflavin T (ThT, catalog# T3516), dimethyl sulfoxide (DMSO, catalog# D8418), BSA (bovine serum albumin, catalog# A3059-100G), RIPA buffer (radio immunoprecipitation assay buffer, catalog# R0278), protease inhibitor (catalog# 11836170001), phosphatase inhibitor (catalog# 04906845001), and 30-kDa molecular weight cut-off filter (catalog# UFC903024) were purchased from Sigma-Aldrich. Primary antibodies used for immunostaining and immunoblotting are as follows: 6E10 (anti-Aβ, catalog# SIG-39320, Covance), GFAP (glial fibrillary acidic protein, catalog# AB5541, Millipore Corporation), Iba-1 (ionized calcium-binding adaptor molecule 1, catalog# MABN92, Millipore Corporation), synaptophysin (catalog# MAB5258, Millipore Corporation), PSD95 (postsynaptic density protein 95, catalog# MA1-046, Invitrogen), total CREB (cAMP response element-binding protein, catalog# sc-186, Santa Cruz Biotechnology, Inc.), p-CREB (catalog# 9198, Cell Signaling Technology, Inc.), total JNK (c-Jun N-terminal kinase, catalog# 9252, Cell Signaling Technology, Inc.), p-JNK (catalog# 9251, Cell Signaling Technology, Inc.), and β-actin (catalog# MAB1501R, Millipore Corporation). Secondary antibodies (HRP-linked IgG) used in immunoblotting analyses were anti-mouse (catalog# A90-116P, Bethyl Laboratories, Inc.), anti-rabbit (catalog# A120-101P, Bethyl Laboratories, Inc.), and anti-goat (catalog# sc-2020, Santa Cruz Biotechnology, Inc.). Fluorescent secondary antibodies (Alexa Fluor 488 and Alexa Fluor 568) were purchased from Invitrogen (catalog# A-28175 and A-11041). We purchased skim milk from Difco (catalog# 232100). PBS (phosphate-buffered saline, pH 7.4, catalog# 11010-023), HBSS (Hank’s balanced salt solution, catalog# 14185-052), trypsin (catalog# 25200-056), DMEM (Dulbecco’s modified eagle medium, catalog# 11995-0685), FBS (fetal bovine serum, catalog# 16000-044), horse serum (catalog# 26050088), neurobasal medium (catalog# 10888-022), B27 supplement (catalog# 12587-010), l-glutamine (catalog# 25030-149), penicillin–streptomycin (catalog# 15140122) were purchased from Gibco. Coverglass was obtained from Marienfeld Superior (catalog# 0117520) and DNase I from Merck & Co. (catalog# 4716728001). ECL solution (SuperSignal West Pico PLUS Chemiluminescent Substrate, catalog# 34580) and BCA assay (Pierce BCA Protein Assay Kit, catalog# 23225) were purchased from Thermo scientific. Deionized water was produced by Milli-Q plus ultrapure water system from Millipore Corporation. 96-well half area black microplate was purchased from Corning, Inc. (catalog# 3694).

### Animals

5XFAD mice (B6SJL-Tg(APPSwFlLon,PSEN1*M146L*L286V)6799Vas/Mmjax) were obtained from the Jackson Laboratory and have been maintained by mating with C57BL/6 × SJL wild type mice. Prior to administration, the genotype of all mice was confirmed by PCR analysis of tail DNA using the standard PCR condition from Jackson Laboratory. All mice were housed in a laboratory animal breeding room in Yonsei University and were maintained under controlled temperature and humidity with an alternating 12-h light–dark cycle and access to food and water ad libitum. For in vivo experiments, 7-month-old male 5XFAD mice were administered with quinacrine (n = 6), and non-treated 5XFAD mice were used (n = 8) as controls.

All animal experiments were performed in accordance with the National Institutes of Health guide for the care and use of laboratory animals and the ARRIVE guidelines. The animal experiment protocols were approved by the Institutional Animal Care and Use Committee of Yonsei University (IACUC-202003-1038-02).

### ThT fluorescence assays

Synthetic Aβ(1–42) peptides were dissolved in DMSO as 5 mM stock^14^, and the quinacrine stock was prepared at 1 mM in DMSO. To examine the ability of quinacrine to inhibit Aβ aggregation, 25 µM of Aβ(1–42) peptides were incubated with different concentrations of quinacrine (0.5, 5, 50 µM) for 3 days at 37 °C, at final DMSO concentration of 5.5% DMSO in deionized water. For Aβ fibril dissociation assays, 5 mM stock of Aβ(1–42) was diluted to 50 µM with deionized water and incubated for 3 days at 37 °C to obtain Aβ aggregates. Different concentrations of quinacrine (1, 10, 100 µM) were prepared by diluting the 1 mM stock in deionized water and incubated with pre-formed Aβ aggregates for 3 additional days at 37 °C. The final Aβ concentration was 25 µM, and quinacrine was 0.5, 5, and 50 µM, with a final DMSO concentration of 5.5% DMSO in deionized water.

After either the inhibition or dissociation incubation was completed, the level of β-sheets of Aβ aggregates was analyzed in the ThT assays^15^. 75 µL of ThT solution (5 µM in 50 mM glycine buffer, pH 8.5) was added to 96-well half area black microplate with 25 µL of incubated Aβ samples. Fluorescence of Aβ-bound ThT was measured at 450 nm (excitation) and 485 nm (emission) using a multimode plate reader (Infinite M200 PRO, Tecan Life Sciences). We also measured the fluorescence intensity of quinacrine at different concentrations (0.5, 5, 50 µM) and deducted it from the fluorescence intensity of samples with Aβ and quinacrine at respective concentrations. Fluorescence intensity was then normalized to incubated Aβ samples without quinacrine treatment (100%) as a control. The results were organized into graphical data with Prism 9, and statistical analyses were performed using one-way ANOVA followed by Bonferroni’s post hoc comparisons (*P < 0.05 and ***P < 0.001). The error bars represent the SD.

### Molecular weight cut-off filtration and dot blot assay

Synthesized Aβ(1–42) was dissolved in DMSO as 10 mM stock and diluted to 100 µM with deionized water^14^, and quinacrine stock was prepared as 10 mM in DMSO. To study whether quinacrine dissociates Aβ fibrils, 100 µM monomeric Aβ(1–42) was incubated for 24 h at 37 °C, followed by incubation with quinacrine for additional 24 h at 37 °C. The final concentration of Aβ was 50 µM, and quinacrine was 100 µM, at a DMSO concentration of 5.5%. The incubated samples were filtered through a 30-kDa molecular weight cut-off filter. For the dot blot assay, 5 µL of the filtrate was spotted onto a nitrocellulose membrane and completely dried. Considering that the filtrate may contain a low Aβ concentration, this process was repeated five times to spot 25 µL of filtrate. Primary anti-Aβ 6E10 antibody (1:1000) and horseradish peroxidase-conjugated secondary antibody (1:10,000) were used to visualize Aβ in the filtrates. Image J was used for blot quantification, and the quantified data was organized and statistically analyzed with Prism 9.

### Intravenous (IV) administration

Quinacrine was injected into the tail vein of 7-month-old male 5XFAD mice once a week, for 6 weeks at 2 mg/kg. The body weight of each animal was measured on the first day of injection and every 7 days afterward.

### Immunostaining

After the administration of quinacrine for 6 weeks, treated and non-treated 5XFAD mice were sacrificed. The brains were perfused with 0.9% saline prior to extraction. The extracted brain was divided into two hemispheres. One hemisphere was lysed for immunoblotting, while the other hemisphere was fixed in 4% paraformaldehyde (pH 7.4) and then immersed in 30% sucrose for cryoprotection. Coronal sections (35 µm) were obtained using a Cryostat (Microm HM 525, Thermo Scientific) and prepared on glass slides. To observe the level of Aβ plaques and astrocytosis, the slides were stained with anti-6E10 monoclonal antibody (1:200 in 5% horse serum) and anti-GFAP polyclonal antibody (1:300 in 5% horse serum). Alexa Fluor 488- and Alexa Fluor 568-conjugated secondary antibodies were used for fluorescence detection. Images were taken on Leica DM2500 fluorescence microscope^16^. Plaque numbers and sizes were quantified using Image J, and the data was organized and statistically analyzed with Prism 9.

### Immunoblotting

The cortical and hippocampal regions were collected separately from the brains of 5XFAD mice. Brain tissues were then homogenized in RIPA buffer with protease inhibitors and phosphatase inhibitors, incubated on ice for 30 min, and centrifuged at 17,000 rpm, at 4 °C for 30 min. The supernatant of brain lysates was obtained, and the concentrations of lysates were quantified by BCA assay. The brain lysates (25 µg) were subjected to SDS–PAGE and transferred to a nitrocellulose membrane. Membranes were blocked with skim milk or bovine serum albumin and treated with primary antibodies overnight at 4 °C. Blots were detected by horseradish peroxidase-conjugated secondary antibodies and developed with the ECL solution following the manufacturer’s instructions. The blots were quantified using Image J and were organized into graphical data with Prism 9 by normalization to β-actin. Statistical analyses were performed using one-way ANOVA followed by Bonferroni’s post hoc comparisons (*P < 0.05, **P < 0.01, ***P < 0.001). The error bars represent the SD.

### Cell treatment

Sprague–Dawley rat postnatal 1 (P1) pups were dissected, and cortex was collected. Rat cortical neurons were prepared as reported previously^17^. Briefly, the cortex was dissected and trypsinized with HBSS containing 0.25% trypsin, 6 mg/mL DNase I for 15 min at 37 °C. The trypsinization was stopped by adding DMEM including 10% FBS and 10% horse serum, and the tissues were triturated and centrifuged. 3 × 10^5^ cells on the coverglass were cultured in a neurobasal culture medium consisting of 2% B27 supplement, 2 mM l-glutamine, and 1X penicillin–streptomycin. Synthesized Aβ(1–42) was dissolved in DMSO at 10 mM and diluted to 1 mM in deionized water (10% DMSO). It was then incubated overnight at 37 °C to produce aggregates prior to treatment. Quinacrine was dissolved in DMSO as a 10 mM stock. To treat Aβ aggregates with or without quinacrine to cortical neurons, we diluted aggregated Aβ and quinacrine stock with neurobasal medium to make 20 µM of Aβ aggregates and 2 µM of quinacrine (0.22% DMSO). Neurons were then treated with 0.22% DMSO as a control, 20 µM of Aβ aggregates, or 20 µM of Aβ aggregates with 2 µM of quinacrine, and they were incubated for 16 to 24 h.

### Cell morphology analysis

Primary cell images were obtained with a CMOS camera (SONY CMOS sensor KOPTIC) using an inverted microscope (Nikon ECLIPSE Ts2). Images were processed with a software (HKBasic 3.7).

### Whole-cell patch recording

Whole-cell patch recording was performed on primary rat cortical neurons. The external solution was continually perfused and composed of (mM): 150 NaCl, 3 KCl, 2 CaCl_2_, 5.5 glucose, and 10 HEPES (pH 7.4 by NaOH; osmolarity adjusted to 315–320 mOsmol/kg with sucrose). The internal solution was 150 CsMeSO_4_, 10 NaCl, 0.5 CaCl_2_, 10 HEPES (pH adjusted to 7.3 with CsOH and osmolarity adjusted to 310 mOsmol/kg with sucrose). EPSC recording was performed in gap-free mode under voltage clamp (V_h_ =  − 70 mV) for 2 min. Prior to EPSC, action potential number was measured under current clamp step (from − 60 to 120 pA, current interval 20 pA, 10 steps). All data were acquired, stored, and analyzed using pCLAMP 10 (Axon Instruments), Digidata 1322, and Mini Analysis Program (Synaptosoft, Inc.). Acceptable access resistances were under 50 MΩ.

### Data analysis

Raw traces were plotted in SigmaPlot 10.0. For analyzing kinetics, Mini Analysis was used (Synaptosoft, Inc.). EPSCs were automatically detected over the threshold of 10 pA. For analyzing EPSC kinetics, currents were detected for 1 min and averaged for each experimental condition from 2 min recording. For statistics, Prism 9 was used for one-way ANOVA Dunnett's post hoc test.

## Results

### Quinacrine dissociates Aβ aggregates

**Figure 1:**
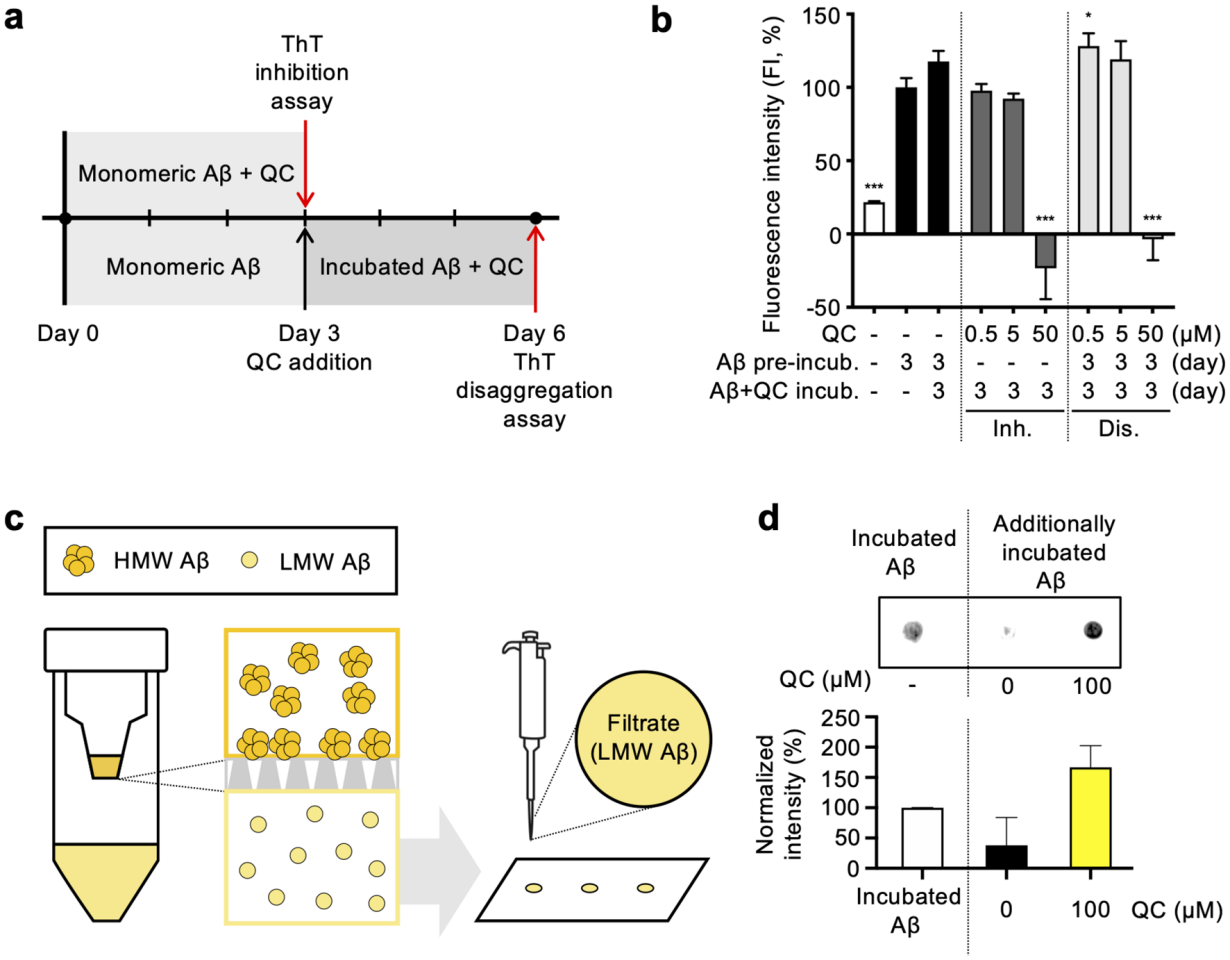
Quinacrine inhibits and reverses Aβ aggregation in vitro. (**a**) The procedure of sample preparation and analyses of ThT assays. In the inhibition assay, monomeric Aβ (25 µM) and quinacrine (0.5, 5, 50 µM) were incubated together for 3 days at 37 °C. For disaggregation assay, monomeric Aβ (50 µM) was incubated for 3 days at 37 °C and further incubated with quinacrine (0.5, 5, 50 µM) for 3 additional days at 37 °C. (**b**) ThT assays for inhibition and disaggregation of Aβ aggregation. After incubation of quinacrine with monomeric or 3-day aggregated Aβ, ThT was added, and the FI was measured. FI was normalized to Aβ aggregates (100%, 3-day). The raw data of ThT inhibition and disaggregation assays are presented in Supplementary Table S1. (**c**) Schematic illustration of molecular weight cut-off (MWCO) filtration and dot blot assay. Synthesized Aβ was incubated for 1 day at 37 °C to produce aggregates, and further incubated for 1 day at 37 °C with quinacrine. The samples were filtered through 30-kDa MWCO filter, and the filtrate was dotted onto a membrane. The level of Aβ in the filtrates was detected by anti-Aβ 6E10 antibody. (**d**) Dot blot assay and quantified data. Pre-formed Aβ aggregates were normalized to 100% as a control. All data are presented as ± SD. *P < 0.05 and ***P < 0.001 (one-way ANOVA followed by Bonferroni’s post hoc comparisons tests). QC, quinacrine; FI, fluorescence intensity; incub., incubation; Inh., inhibition assay; Dis., disaggregation assay; HMW, high-molecular-weight; LMW, low-molecular-weight.

To examine the inhibitory effect of quinacrine on Aβ aggregation, we incubated monomeric Aβ(1–42) (25 µM) with or without quinacrine (0, 0.5, 5, and 50 µM) in aqueous environments for 3 days at 37 °C, and the level of Aβ fibril formation was measured utilizing ThT, a fluorescent chemical intercalating into the β-sheet structures of aggregated Aβ^15^. The ThT-detected level of Aβ fibril formation in the incubated samples was then normalized by the fluorescence intensity (FI) of 3-day incubated Aβ (Aβ_3-day_) as a negative control (Fig. 1a). The FI of quinacrine alone (QC_3-day_) was deducted from the FI of aggregated Aβ with quinacrine ((QC + Aβ)_3-day_) to exclude quinacrine-associated fluorescence signal. The final FI (%) was calculated with the following equation, in which the subscripts indicate the length of incubation period:

1 $$FI\left(\%\right)=\frac{{FI\left(QC+A\beta \right)}_{3 {\text{-}}day}-{FI(QC}_{3{\text{-}}day})}{{FI(A\beta }_{3{\text{-}}day})}\times 100$$

While samples with 0.5 and 5 µM quinacrine had similar FI as to Aβ_3-day_, 50 µM of quinacrine induced a significant decrease in ThT fluorescence signals (Fig. 1b). This indicates that quinacrine substantially prevented the formation of Aβ fibrils. Next, to examine whether quinacrine dissociates Aβ aggregates, we incubated Aβ for 3 days to obtain Aβ fibrils (Aβ_3-day_) in advance and, then, incubated for 3 additional days with or without quinacrine (0, 0.5, 5, and 50 µM) at 37 °C. ThT assays were conducted and FI was normalized by the FI of Aβ_3-day_ (Fig. 1a). The final FI (%) was calculated with the following equation:

2 $$FI\left(\%\right)=\frac{{FI \left(QC+{A\beta }_{3{\text{-}}day}\right)}_{3{\text{-}}day}-{FI(QC}_{3{\text{-}}day})}{{FI(A\beta }_{3{\text{-}}day})}\times 100$$

Samples with 0.5 and 5 µM of quinacrine had increased FI after the additional 3-day incubation, while Aβ with 50 µM of quinacrine induced a significant drop in ThT FI to a negative value (Fig. 1b). An ideal dissociation of fibrils shall result in 0% FI results, and the negative value of 50 µM quinacrine-treated sample is due to the larger FI(QC_3-day_) than the FI(QC + Aβ_3-day_)_3-day_ as quinacrine is a fluorescence chemical of its excitation and emission spectra (λ_ex_ = 436 nm/λ_em_ = 492 nm) overlapping with those of ThT (λ_ex_ = 450 nm/λ_em_ = 485 nm)^18,19^.

Thus, we designed another set of in vitro experiments excluding possible interference of quinacrine on assay results due to its color by physically separating dissociated Aβ from fibrils upon MWCO filtration (Fig. 1c). Monomeric Aβ(1–42) (100 µM) was incubated for 1 day to obtain the aggregated form (Aβ_1-day_), which was then co-incubated with or without 200 µM of quinacrine for 1 additional day. Quinacrine-treated or non-treated Aβ samples were filtered through a 30-kDa MWCO filter, and the soluble and relatively smaller Aβ species found in the filtrate was considered as low-molecular-weight (LMW) Aβ. The LMW Aβ in the filtrates was detected by anti-Aβ 6E10 antibody in dot blot assays. For densitometric analyses, the intensity of Aβ_1-day_ dot spot was used as a control (100%) for data normalization. As a result, in the filtrate of Aβ aggregates without quinacrine, LMW Aβ was barely detectable as Aβ_1-day_ further accumulated into high-molecular-weight (HMW) Aβ aggregates, larger than 30 kDa. On the other hand, the filtrate of quinacrine-treated Aβ sample displayed a stronger dot signal in comparison to that of Aβ_1-day_, indicating that the sample with quinacrine contains a greater amount of LMW Aβ (Fig. 1d). These results provide evidence and support our ThT assay results that quinacrine directly dissociates pre-formed Aβ aggregates.

### Quinacrine reverses Aβ deposition and synaptic abnormality in 5XFAD

**Figure 2:**
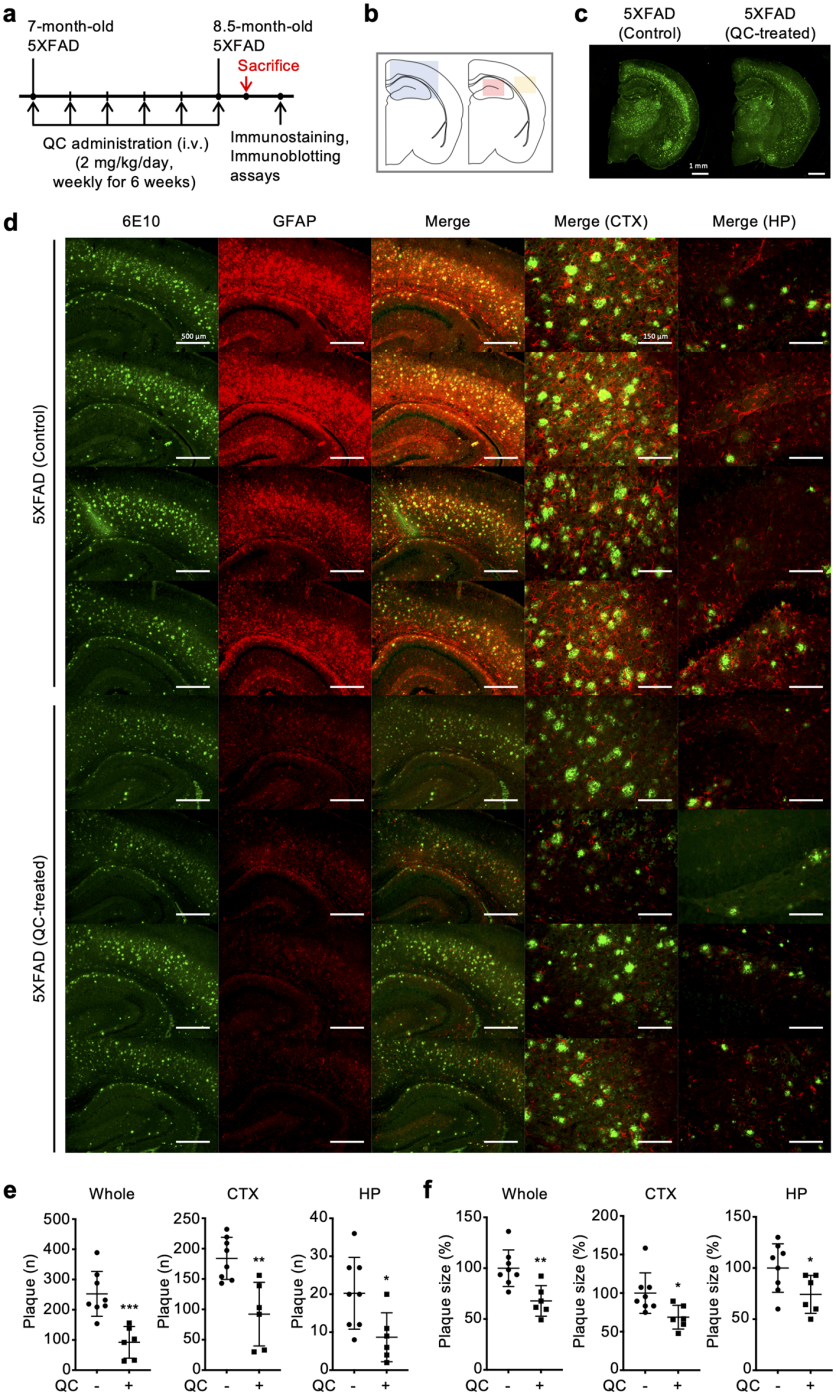
Quinacrine reduces Aβ plaques and astrocytosis. (**a**) Time course of the in vivo experiments. Quinacrine was administered for 6 weeks intravenously, and the brains were collected for immunostaining and immunoblotting assays. (**b**) The colored boxes indicate the regions of brain images in (**d**). Blue: regions of brain images in lanes 1, 2, 3 in (**d**); red: HP; yellow: CTX. (**c**) Representative immunostaining images of Aβ deposition. 6E10 antibody was used for the visualization of Aβ plaques. Scale bars = 1 mm. (**d**) Representative immunostaining images of Aβ deposition and astrocytosis. Aβ was stained with the 6E10 antibody, and the level of astrocytosis was monitored using GFAP antibody. CTX and HP merge images are enlarged images of respective brain regions. Scale bars = 500 µm, 150 µm. (**e**) Number of plaques and (**f**) plaque size in whole brain area, cortex, and hippocampus. The plaque sizes of non-treated mice brains were normalized to 100%. Data are presented as ± SD. *P < 0.05, **P < 0.01, and ***P < 0.001 (one-way ANOVA followed by Bonferroni’s post hoc comparisons tests). QC, quinacrine; i.v., intravenous injection; CTX, cortex; HP, hippocampus.

To evaluate the dissociation function of quinacrine against aggregated Aβ in vivo, we utilized a 5XFAD transgenic AD mouse model. As this mouse model begins to develop Aβ deposition in the brain at 2 months of age, we used 7-month-old male 5XFAD mice, in which Aβ plaques are highly accumulated^20^. Quinacrine was intravenously injected for 6 weeks (2 mg/kg, weekly, n = 6), and non-treated mice were prepared as controls (n = 8). After 6 weeks, mouse brains were collected and divided into hemispheres, each of which was prepared as either brain slides or lysates to examine the levels of Aβ plaques and the subsequent alterations in the expression of AD-related proteins (Fig. 2a). To visualize plaques in the brain, an anti-Aβ 6E10 antibody was used, and the number and size of Aβ plaques were quantified in the cortex and hippocampus, separately (Fig. 2b–d). We observed a significantly reduced amount of Aβ plaques in quinacrine-treated mice compared to the control, with over a two-fold decrease in plaque numbers (Fig. 2e). We also found that the average size of plaques in the quinacrine-treated group was significantly smaller than the control, implying Aβ plaque dissociation by quinacrine administration (Fig. 2f). Furthermore, astrocytosis, an Aβ-associated inflammatory reaction, was examined through the histochemical imaging of glial fibrillary acidic protein (GFAP) expression^21–23^. Compared to the control, we observed reduced GFAP levels in the brains of quinacrine-treated 5XFAD mice along with a significant reduction of Aβ plaques in the brain (Fig. 2d).

**Figure 3:**
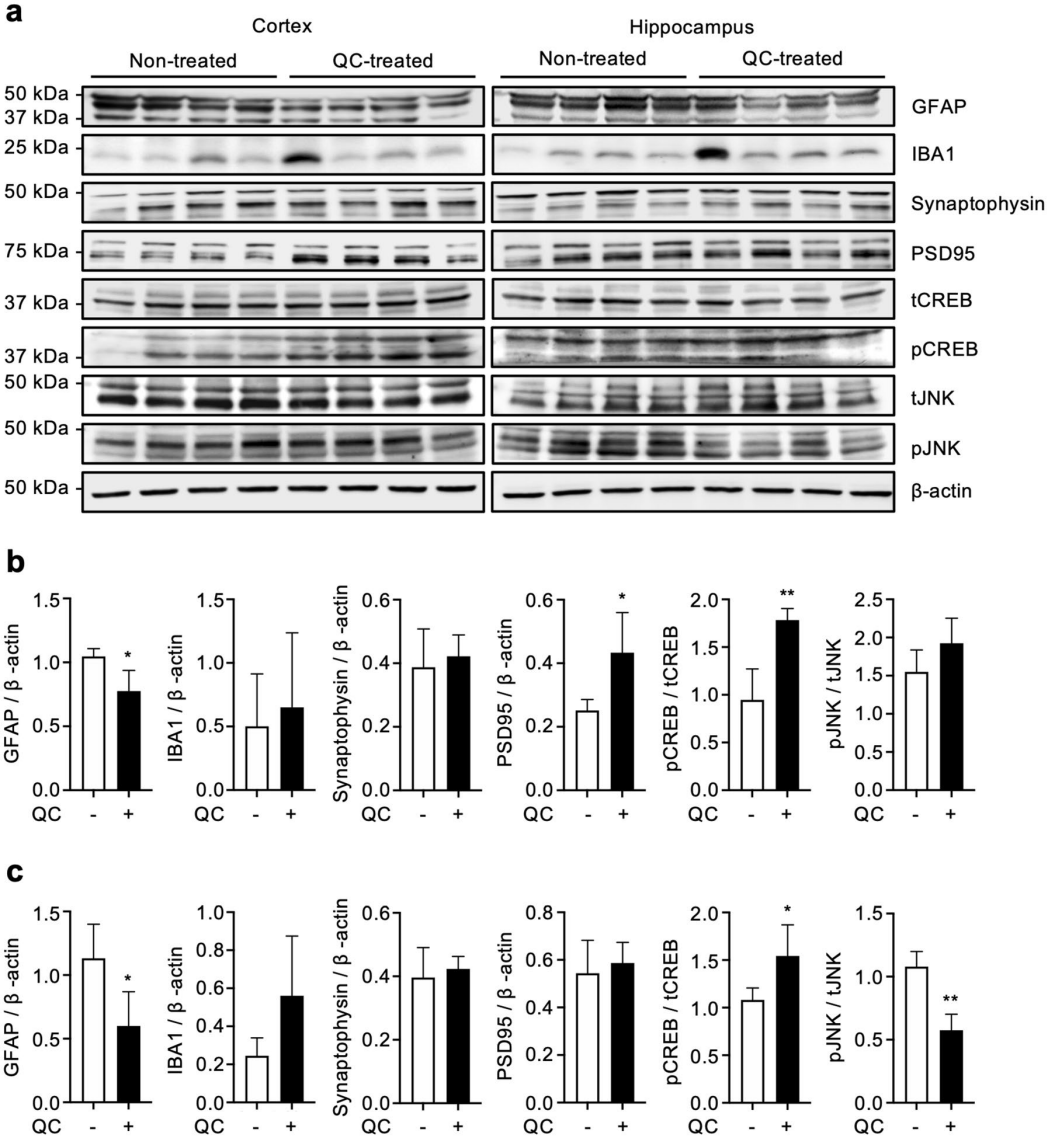
Quinacrine ameliorates the expression levels of AD-related biomarkers. (**a**) Western blot and (**b**) densitometry analysis of GFAP, Iba-1, synaptophysin, PSD95, phosphorylated CREB (pCREB) and JNK (pJNK) expressions in cortical and (**c**) hippocampal lysates. The full-membrane images and cropped membranes with respective β-actins are presented in Supplementary Figures S2 and S3. Data are presented as ± SD. *P < 0.05 and **P < 0.01 (Unpaired t-test). QC, quinacrine; GFAP, glial fibrillary acidic protein; Iba-1, ionized calcium-binding adaptor molecule 1; PSD95, postsynaptic density protein 95, CREB, cAMP response element-binding protein; JNK, c-Jun N-terminal kinase.

Using the other hemisphere, we assessed the altered levels of astrocytosis and microgliosis by western blots detecting GFAP and ionized calcium-binding adaptor molecule 1 (Iba-1), respectively (Fig. 3a–c). Also, the expression levels of synaptophysin and postsynaptic density protein 95 (PSD95) were examined as markers of pre- and post-synaptic density protein, respectively. In addition, we studied the phosphorylation levels of cyclic AMP response element-binding protein (CREB) and c-Jun N-terminal kinase (JNK), which are associated with long-term synaptic plasticity^24^ and long-term potentiation dysfunction^25^, respectively. Consistent with the immunostaining results, we observed that quinacrine treatment significantly lowered the level of GFAP in both cortex and hippocampus. On the other hand, we could not find any changes upon the quinacrine treatment in Iba-1 expression levels. While synaptophysin was expressed at a similar level in both control and quinacrine-treated mice, PSD95 expression was significantly upregulated in the cortex of the quinacrine-treated mice. In the quinacrine-treated group, levels of phospho-CREB increased in both cortex and hippocampus, and phospho-JNK level decreased in the hippocampal region. Collectively, these results demonstrate that quinacrine administration lowers the level of Aβ plaques in 5XFAD mice, leading to reduction of Aβ-associated astrocytosis, and also suggest the amelioration of synaptic dysfunction by quinacrine administration.

### Dissociation of Aβ aggregates by quinacrine ameliorates Aβ-induced synaptic dysfunction

**Figure 4:**
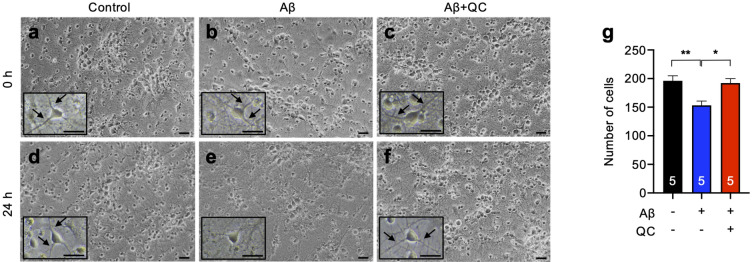
Quinacrine rescues Aβ-mediated loss of cell number and neuronal processes. (**a**–**f**) Representative images of 0-h and 24-h incubated neurons after treatment of control (DMSO), Aβ, or Aβ + QC. Evident loss of neuronal processes was observed 24 h after neurons were treated with Aβ. Scale bars = 20 µm. The insets are enlarged images (×400), and the arrows indicate normal processes. Scale bars = 10 μm. (**g**) Number of cells in captured images (×200). Aβ + QC-treatment significantly reduced the level of Aβ-induced cell death. The cells were counted from five fields of view for each indicated condition (n = 5, each condition). Data are presented as ± SEM. *P < 0.05, **P < 0.01 (one-way ANOVA followed by Tukey’s post hoc test). QC, quinacrine.

To investigate the protective effects of quinacrine against Aβ-induced synaptic abnormalities, we measured EPSC from primary cortical neurons isolated from Sprague–Dawley rat pups (postnatal day 1)^17^. Aβ aggregates (20 µM) were treated to the primary neurons with or without 2 µM of quinacrine prior to addition to these neurons. When we monitored the alteration of cell morphology 24 h after the treatment of Aβ or Aβ with quinacrine (Aβ + QC), we observed markedly shortened neuronal processes and the reduced cell number in Aβ-treated cells, whereas the morphology and the number of Aβ + QC-treated cells were similar to those of the control (DMSO-treated) (Fig. 4a–g).

**Figure 5:**
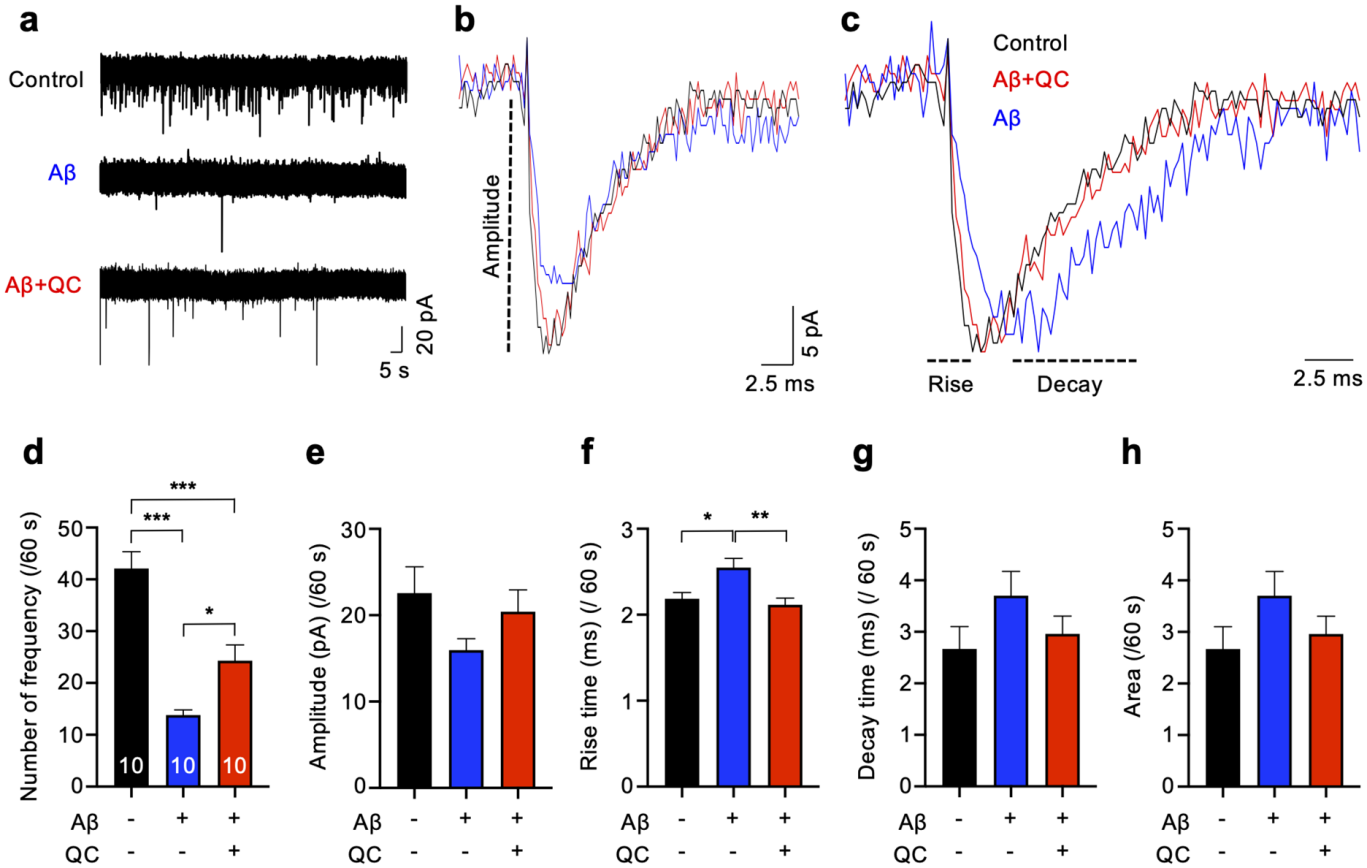
Quinacrine treatment recovers Aβ-induced abnormality of synaptic function. (**a**) Traces for EPSC with control (DMSO, black), Aβ (blue), and Aβ + QC-treated cells (red). Scale bars = 5 s, 20 pA for each indications. (**b**) Averaged traces, black for control, blue for Aβ and red for Aβ + QC. Scale bars = 2.5 ms, 5 pA for each indication. (**c**) Normalized traces from average (divided by peak amplitude) for the analyses of rise and decay times. Scale bar = 2.5 ms. (**d**) Summary of EPSC frequency. Quinacrine treatment promotes recovery of Aβ-induced reduction of EPSC frequency. n = 10 for each group. (**e**) Summary of EPSC amplitude. (**f**) Summary of EPSC rise time. (**g**) Summary of EPSC decay time. (H) Summary of EPSC area of each group. Data are presented as ± SEM. *P < 0.05, **P < 0.01, ***P < 0.001 (one-way ANOVA followed by Tukey’s post hoc test). QC, quinacrine.

Furthermore, we measured the EPSC frequency, amplitude, rise time, decay time, and area to analyze alterations in the synaptic function of primary neurons in response to the Aβ-dissociating function of quinacrine (Fig. 5). Aβ treatment induced a lower frequency and longer rise time of EPSC compared to the control (Fig. 5a,c,d,f). These results indicate impaired presynaptic glutamate release and delayed activation of excitatory postsynaptic channel, respectively. We found that Aβ treatment to primary neurons did not alter the amplitude, decay time, and area of EPSCs (Fig. 5b,c,e,g,h). We also observed that Aβ did not alter the number of action potentials, which is an intrinsic property of neuronal excitability (Supplementary Fig. S4). In Aβ + QC-treated cells, we observed higher EPSC frequency compared to the Aβ-treated cells, and the rise time of EPSC in Aβ + QC-treated cells was comparable to the control (Fig. 5a,c,d,f), suggesting that quinacrine treatment protects primary neurons from Aβ-induced presynaptic and postsynaptic dysfunctions. These results demonstrate that Aβ dissociation by quinacrine treatment ameliorates synaptic damages induced by Aβ aggregates.

## Discussion

Here, we report that quinacrine dissociates Aβ plaques, reducing inflammation in 5XFAD mouse brain, and ameliorates Aβ-induced synaptic impairments in primary neurons. While quinacrine was widely used as an antimalarial agent^10^, studies suggest that quinacrine has other promising therapeutic potentials for bacterial infections, inflammatory diseases, and neurodegenerative disorders^26–28^. In this study, we examined the possible anti-aggregation function of quinacrine on Aβ. We were concerned that the vivid yellow color of quinacrine might affect the in vitro results as ThT assays heavily depend on the fluorescence spectrum and intensity of the tested compound. However, immunostaining results of quinacrine-administered 5XFAD mouse brains showed that the numbers and sizes of Aβ plaques significantly decreased throughout the brain when compared to the non-treated group. This indicates that quinacrine dissociates Aβ plaques in the brains of 5XFAD mouse model, which further supports the results of ThT assays to be a demonstration of successful dissociation of Aβ aggregates. Interestingly, we found that there are inconsistencies in the expression levels of synapse-related proteins between cortex and hippocampus, even though immunostaining results showed a similar level of quinacrine-mediated dissociation of Aβ plaques in both regions. These differences in PSD95, phosphorylated JNK and CREB levels are possibly due to the dosage or administration period. In addition, quinacrine treatment prevented Aβ-induced presynaptic damage in primary neurons, whereas the level of a presynaptic marker, synaptophysin, was not affected by quinacrine administration to 5XFAD mice. While it may be due to a time lag between the alteration of protein expression levels and consequent changes in synaptic function, the exact mechanism underlying this result needs to be studied.

During AD pathogenesis, Aβ accumulation starts approximately two decades before the onset of symptom^29^. Consequently, it is extremely difficult to diagnose AD patients at an early stage, increasing the need for AD drug which dissociates pre-existing Aβ plaques. The long use of quinacrine as an antimalarial agent on humans demonstrates its low toxicity and ensures the absence of severe adverse effects^10^, making its application as AD therapeutics easier. Given the recovery of synaptic function in cultured neurons and plaque reduction in 5XFAD brains by quinacrine treatment, further studies on short- and long-term effects of quinacrine treatment for the drug repositioning for AD are warranted.

## Supplementary Information

Supplementary Information.
