# Supplementary material for: Quinacrine directly dissociates amyloid plaques in the brain of 5XFAD transgenic mouse model of Alzheimer’s disease
Source: Sci Rep. 2021 Jun 8;11:12043. doi: 10.1038/s41598-021-91563-y (PMC8187640; doi:10.1038/s41598-021-91563-y)
Supplement: Supplementary file 1 — Supplementary Information. [file 41598_2021_91563_MOESM1_ESM.pdf]

Quinacrine directly dissociates amyloid plaques in the brain of 5XFAD transgenic mouse model of Alzheimer's disease

Sohui Park[1,#], Hye Yun Kim[1,#], Hyun-A Oh[2,#], Jisu Shin[1], In Wook Park[1], Soljee Yoon[1], Dong Ho Woo[2], and YoungSoo Kim[1,*]

[1]*Department of Pharmacy, Department of Integrative Biotechnology and Translational Medicine, and Yonsei Institute of Pharmaceutical Sciences, Yonsei University, Incheon 21983, Republic of Korea*

[2]*Research Center for Convergence Toxicology, Korea Institute of Toxicology, Daejeon 34114, Republic of Korea*

[#]These authors contributed equally to this work.

[*]y.kim@yonsei.ac.kr

	Fluorescence intensities			Mean
$A\beta_{0\text{-day}}$	434	450	459	448
$A\beta_{3\text{-day}}$	1883	1902	2105	1963
$A\beta_{6\text{-day}}$	2309	2165	2442	2305
0.5 μM $QC_{3\text{-day}}$	111	78	96	95
5 μM $QC_{3\text{-day}}$	693	739	815	749
50 μM $QC_{3\text{-day}}$	8990	8630	8977	8866
$(0.5\ \mu M\ QC\ \&\ A\beta)_{3\text{-day}}$	2079	1991	1892	1987
$(5\ \mu M\ QC\ \&\ A\beta)_{3\text{-day}}$	2402	2562	2642	2535
$(50\ \mu M\ QC\ \&\ A\beta)_{3\text{-day}}$	8425	8630	8183	8413
$(0.5\ \mu M\ QC\ \&\ A\beta_{3\text{-day}})_{3\text{-day}}$	2733	2603	2388	2575
$(5\ \mu M\ QC\ \&\ A\beta_{3\text{-day}})_{3\text{-day}}$	3213	3088	2862	3054
$(50\ \mu M\ QC\ \&\ A\beta_{3\text{-day}})_{3\text{-day}}$	9212	8521	8652	8795

Supplementary Table S1. Raw data of ThT inhibition and disaggregation assays. After the addition of ThT to the samples, fluorescence intensity was measured at 450 nm (excitation) and 485 nm (emission). Fluorescence intensity of quinacrine at different concentrations was also measured and deducted from the fluorescence intensity of samples with Aβ and quinacrine. The unit of fluorescence intensity is an arbitrary unit.

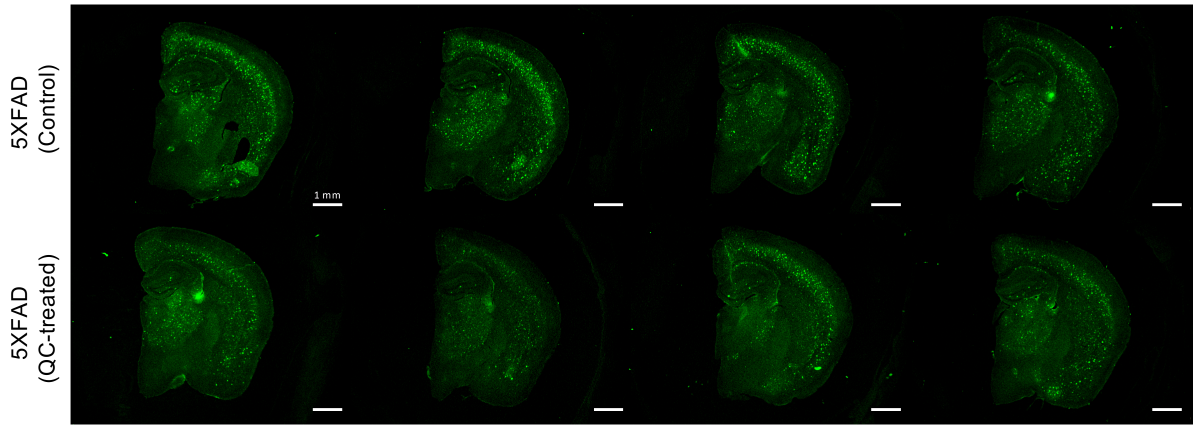

Supplementary Figure S1. Immunostaining images of Aβ deposition. Aβ plaques in the brains of quinacrine-treated and non-treated (control) 5XFAD were visualized with 6E10 antibody.

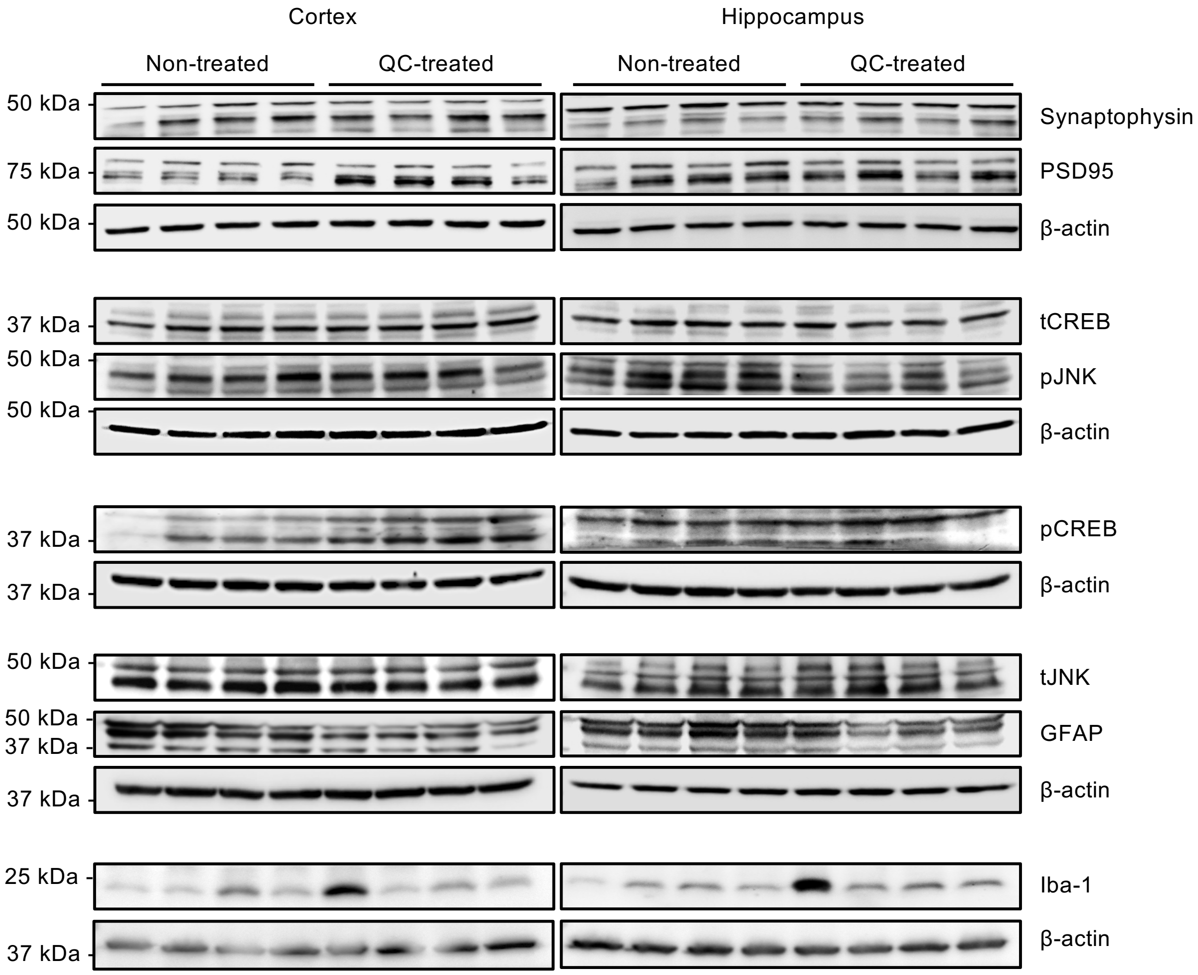

Supplementary Figure S2. Immunoblot results with respective β-actin. The expression levels of multiple markers were observed in the same blot. Synaptophysin and PSD95, tCREB and pJNK, pCREB, tJNK and GFAP, and Iba-1 were blotted on separate membranes.

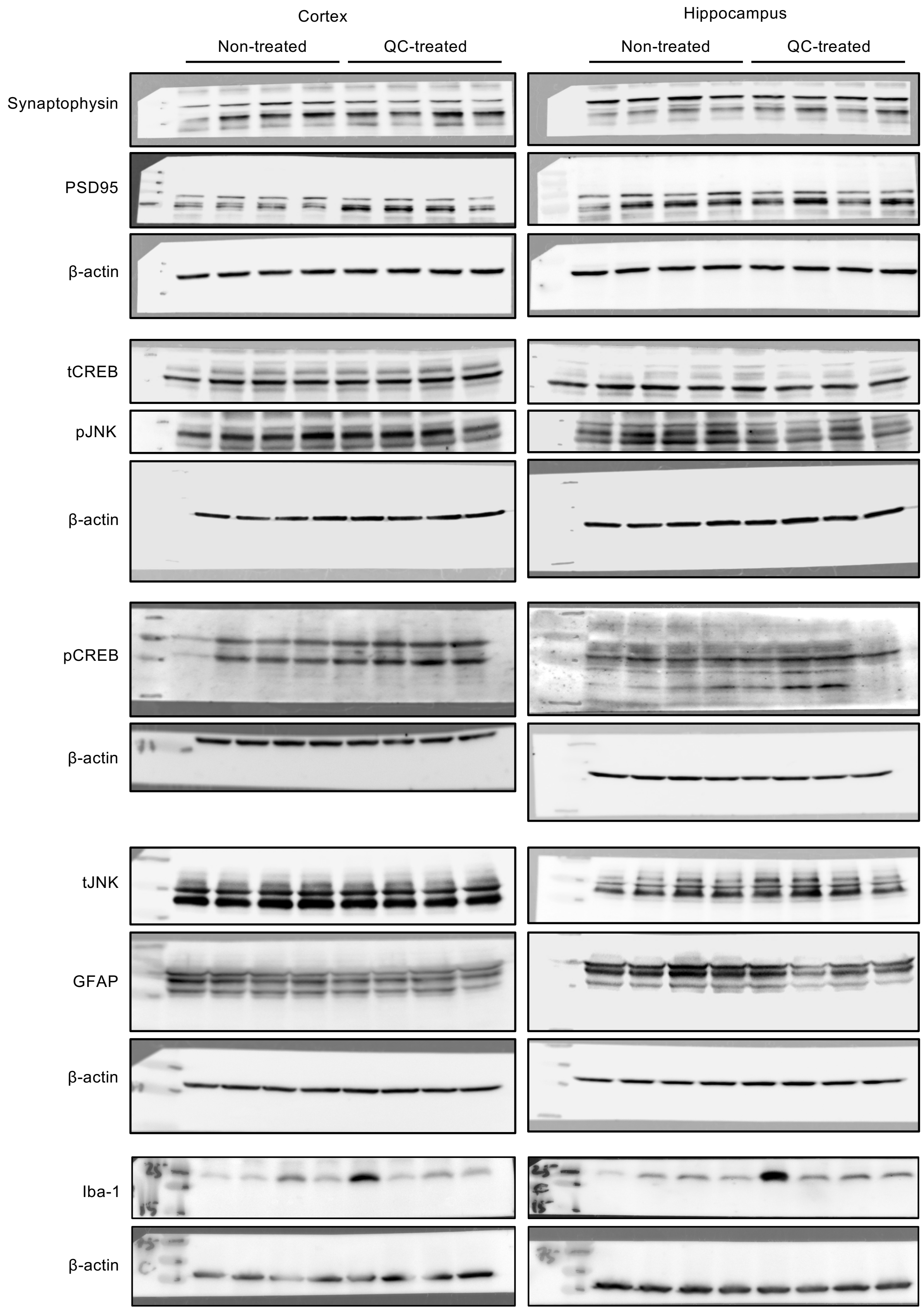

Supplementary Figure S3. Full-membrane images of immunoblots. The full-membrane images are presented in the order of blots presented in Supplementary Figure S2.

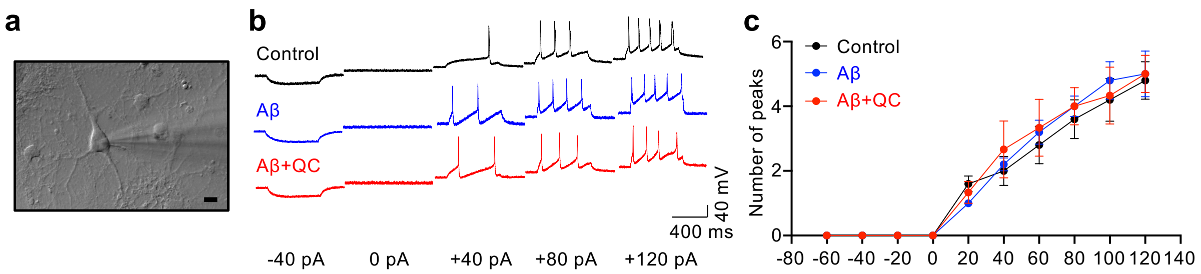

Supplementary Figure S4. The effect of quinacrine and Aβ on the number of action potential in rat cortical neurons. (a) Image depicting the process of whole cell patch recording showing rat cortical neuron with a glass pipette, scale bar = 100 µm. (b) Traces for number of action potential of voltages with current steps −40, 0, 40, 80 and 120 pA. Scale bars = 400 ms, 40 mV for each indication. Black, control; blue, Aβ; red, Aβ+QC. (c) Summary bar for number of action potentials on the indicated current injection in control, Aβ and Aβ+QC groups (n=5 for each group). QC, quinacrine.
